# IgE-mediated food allergy

**DOI:** 10.1186/s13223-018-0284-3

**Published:** 2018-09-12

**Authors:** Susan Waserman, Philippe Bégin, Wade Watson

**Affiliations:** 1 Division of Clinical Immunology and Allergy, Department of Medicine, McMaster University, Hamilton, ON Canada; 2 Division of Clinical Immunology and Allergy, Department of Medicine, Université de Montréal, Montreal, QC Canada; 3 IWK Health Centre, Division of Allergy, Department of Pediatrics, Dalhousie University, Halifax, NS Canada

## Abstract

Food allergy is defined as an adverse immunologic response to a food protein. Food-related reactions are associated with a broad range of signs and symptoms that may involve any body system, including the skin, gastrointestinal and respiratory tracts, and cardiovascular system. Immunoglobulin E (IgE)-mediated food allergy is a leading cause of anaphylaxis and, therefore, referral to an allergist for timely and appropriate diagnosis and treatment is imperative. Diagnosis entails a careful history and diagnostic tests, such as skin prick tests, serum-specific IgE and, if indicated, an oral food challenge. Once the diagnosis of food allergy is confirmed, strict elimination of the offending food allergen from the diet is generally necessary; however, in the case of cow’s milk and egg allergy, many allergic children are able to eat these foods in their baked form. This article provides an overview of the epidemiology, pathophysiology, diagnosis, and management of IgE-mediated food allergy.

## Background

IgE-mediated food allergy is a leading cause of anaphylaxis, a severe, potentially fatal allergic reaction presenting to emergency departments [1] (see article on *Anaphylaxis* in this supplement). A recent survey of over 5700 Canadian households (15,022 individuals) estimated the prevalence of food allergy in Canada to be 7.5% (self-reported; Table 1) [2]. Annually, approximately 200 deaths in the United States are attributed to food allergy [3]. A review of anaphylaxis deaths that occurred between 1986 and 2011 in Ontario, Canada, attributed 48% of these deaths to food allergy [4]. When comparing the time periods 1986–1998 and 1999–2011, fatalities due to food allergy declined from 28 to 12 cases, whereas fatalities due to medications and unknown causes increased (from 6 to 10 and from 1 to 5, respectively).

**Table 1:** Prevalence (self-reported, unadjusted) estimates for probable food allergy in Canada [2]

Food allergen	Prevalence (%)	
	Children	Adults
Peanut	2.2	0.6
Tree nuts	1.5	1.0
Fish	0.9	0.5
Shellfish	0.8	1.6
Sesame	0.1	0.2
Milk	0.2	0.2
Egg	1.0	0.5
Wheat	0.2	0.2
Soy	0.1	0.1

Accurate diagnosis and appropriate management of IgE-mediated food allergy are critical since accidental exposure to even minute quantities of the culprit food may result in anaphylaxis [5]. This review focuses primarily on IgE-mediated food-allergic reactions, and provides an overview of current literature related to the epidemiology, pathophysiology, diagnosis, and management of this type of food allergy.

## Definition

The term food allergy is used to describe an adverse immunologic response to a food protein. It is important to distinguish food allergy from other non-immune-mediated adverse reactions to foods, particularly since more than 20% of adults and children alter their diets due to perceived food allergy [6]. Adverse reactions that are not classified as food allergy include food intolerances secondary to metabolic disorders (e.g., lactose intolerance), reactions to toxic contaminants (e.g., bacteria in decomposing scombroid fish will convert histidine, an amino acid, to histamine) or pharmacologically active food components (e.g., caffeine in coffee causing jitteriness, tyramine in aged cheeses triggering migraine).

## Pathophysiology

Although food allergy can arise to any food, Health Canada has identified the following 10 priority allergens: cow’s milk (CM), egg, peanut, tree nuts, fish/shellfish, wheat, sesame seed, soy, mustard and sulphites (a food additive) [7]. Canadian food labeling regulations require food manufacturers to list these food allergens, gluten sources and added sulphites on food labels.

With the exception of a carbohydrate known as galactose-alpha-1,3-galactose (also known as alpha-gal), it is the protein component, not the fat or carbohydrate components, of these foods that leads to sensitization and allergy. The allergenic segments or epitopes of these proteins tend to be small (10–70 kd in size), water-soluble glycoproteins that have varying degrees of resistance to denaturation by heat or acid and, therefore, can remain intact even after processing, storage, cooking and digestion [5, 6, 8]. Examples of these glycoproteins include casein in CM, vicilin in peanut, and ovomucoid in egg. However, many children who are allergic to CM and egg can tolerate these foods when baked. In general, allergies to additives and preservatives are uncommon.

IgE antibody responses to alpha-gal results in a delayed allergic reaction to mammalian meat, and has been associated with anaphylaxis 3–6 h after ingestion of mammalian food products (e.g., beef and pork) [9]. It is the only example of IgE to a carbohydrate that has been associated with anaphylaxis. Studies strongly suggest that tick bites are the main cause of this IgE antibody response to alpha-gal, since ticks inject alpha gal through their saliva when biting humans [9].

Food-induced allergic disorders are broadly categorized into those mediated by IgE antibodies or by non-IgE-mediated mechanisms. IgE-mediated allergic responses are the most widely recognized form of food allergy and are characterized by the rapid onset of symptoms after ingestion. During initial “sensitization” to the food, consumption of the allergenic food protein stimulates production of food-specific IgE antibodies which then bind to tissue basophils and mast cells. When the causal foods are subsequently eaten, they bind to their specific IgE antibodies and trigger the release of mediators, such as histamine, prostaglandins and leukotrienes, causing “clinical reactivity” (allergic symptoms). It is important to note that sensitization can be present *without* clinical reactivity, meaning that specific IgE to a food is present, but no reaction occurs with food exposure [6, 8, 10, 11]. For a review of non-IgE-mediated (cell-mediated) food hypersensitivity, please see the article entitled *Non*-*IgE*-*mediated Food Hypersensitivity* in this supplement.

Disorders such as atopic dermatitis (AD), eosinophilic gastroenteritis, and eosinophilic esophagitis (EoE) may be associated with a mixed IgE-/cell-mediated mechanism of reactivity to food (see articles on EoE and AD in this supplement). In these disorders, the association with food may not be demonstrated in all patients.

**Fig. 1:**
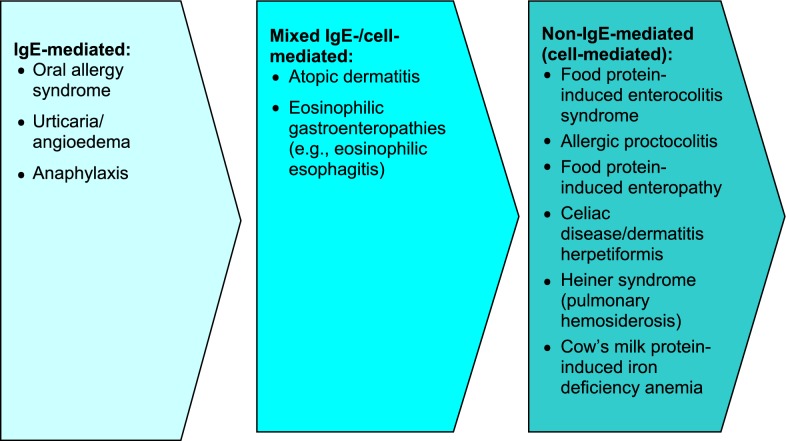
**Spectrum of food allergy disorders according to pathophysiology** [6, 8, 10]

The spectrum of food-allergy-associated disorders according to pathophysiology is shown in Fig. 1. It is important to note that food allergy is not a cause of conditions such as migraines, behavioural or developmental disorders, arthritis, seizures or inflammatory bowel disease.

## Natural history of food allergy

The natural history of food allergy varies by type of food allergen. CM and egg allergy can present in the 1st year of life, and although some children may outgrow these allergies by early school age, others may not develop tolerance until their teenage years. Studies have reported that 19% of subjects achieve tolerance to CM by age 4 years, 42% by age 8 years, 64% by age 12 years, and 79% by 16 years [12]. For egg allergy, 4% achieve tolerance by age 4 years, 12% by age 6 years, 37% by age 10 years, and 68% by age 16 years [13]. In contrast, allergy to peanut, tree nuts, fish, and shellfish are generally lifelong, although 20% of individuals may outgrow peanut allergy [14]. Peanut and tree nuts are responsible for the most serious allergic reactions and food-allergy related fatalities [15, 16].

Children with AD tend to have a higher prevalence of other atopic disorders including food allergy. Approximately 35% of children with moderate-to-severe AD have IgE-mediated food allergy [17], as well as a higher prevalence of allergic rhinitis (75%) and asthma (80%) [18]. Primary prevention in infants at increased risk of AD is discussed in this supplement (see *Atopic Dermatitis* article).

## Clinical manifestations

Food-related reactions are associated with a broad range of signs and symptoms that may involve any body system, including the skin, GI and respiratory tracts, and cardiovascular system (Table 2). Food allergy is not felt to play a role in chronic respiratory symptoms.

**Table 2:** Signs and symptoms of food allergy

	IgE-mediated (immediate reactions)	Non-IgE-mediated (delayed/chronic reactions)
**Skin**		
Urticaria	√	
Angioedema	√	
Erythema	√	√
Pruritus	√	√
Eczematous rash/lesions	√	√
**Respiratory**		
Laryngeal edema	√	
Rhinorrhea	√	
Bronchospasm	√	
Nasal congestion	√	
Cough	√	
Chest tightness	√	
Wheezing	√	
Dyspnea	√	
**Gastrointestinal**		
Angioedema of the lips, tongue, palate	√	
Oral pruritus	√	
Tongue swelling	√	
Vomiting	√	√
Diarrhea	√	√
Pain	√	√
**Cardiovascular**		
Presyncope/syncope	√	
Hypotension	√	
Tachycardia	√	

Skin reactions, including urticaria, angioedema and erythema, are the most common clinical manifestations of IgE-mediated allergy to food. Typical respiratory symptoms include laryngeal edema, rhinorrhea, and bronchospasm. GI-related signs and symptoms of food allergy include nausea, vomiting, abdominal pain, and diarrhea.

A localized IgE-mediated reaction is the oral allergy syndrome, also known as the pollen-food syndrome, which causes tingling and itching of the mouth and pharynx. This is typically triggered after consumption of certain fresh fruits and vegetables in pollen-allergic individuals. It is caused by cross reactivity between IgE antibodies to certain pollens with proteins in some fresh fruits and vegetables (see Table 3) [5]. For example, individuals with ragweed allergy may experience oropharyngeal symptoms following the ingestion of bananas or melons, and patients with birch pollen allergy may experience these symptoms following the ingestion of raw carrots, celery or apple. Fortunately, these proteins are heat labile, enabling allergic individuals to eat these foods when cooked. Allergy skin tests are usually negative to commercial food extracts in individuals with oral allergy syndrome, but are positive to the fresh or frozen food [19]. Also, progression to systemic symptoms is rare, but may occur in a small proportion of patients with this condition [6, 8, 20].

**Table 3:** Oral allergy syndrome: cross reaction between proteins in pollen and fresh fruits and vegetables [5]

Pollen	Fresh fruit/vegetable/nuts	
Birch	• Almond	• Kiwi
	• Apple	• Nectarine
	• Apricot	• Peach
	• Brazil nut	• Peanut
	• Carrot	• Pear
	• Celery	• Plum
	• Cherry	• Potato
	• Coconut	• Swede
	• Fennel	• Tomato
	• Hazelnut	• Walnut
Ragweed	• Banana	• Honeydew
	• Cantaloupe	• Watermelon
	• Cucumber	• Zucchini
Grass	• Cherry	• Peach
	• Kiwi	• Potato
	• Orange	• Tomato
	• Melon	• Watermelon

The most severe reaction is anaphylaxis, which is defined as a serious allergic reaction that is rapid in onset and may cause death. The clinical criteria for diagnosing anaphylaxis are shown in Table 4 [21–23]. There are numerous signs and symptoms of anaphylaxis which usually develop within minutes to 2 h after food exposure. Early symptoms should not be ignored since reactions can be highly unpredictable, and can vary from person to person, and even from attack to attack in the same person. Peanuts, tree nuts, shellfish, fish, CM, and eggs are the most common foods that cause anaphylaxis; however, any food can trigger an allergic reaction [5].

**Table 4:** Clinical criteria for diagnosing anaphylaxis [21–23]

Anaphylaxis is highly likely when any 1 of the following 3 criteria is fulfilled following exposure to an allergen
1. **Acute onset of an illness** (minutes to several hours) **with involvement of the skin, mucosal tissue, or both** (e.g., generalized hives, pruritus or flushing, swollen lips-tongue-uvula) **and at least 1 of the following**: a. **Respiratory compromise** (e.g. dyspnea, wheeze, bronchospasm, stridor, reduced PEF, hypoxemia) b. **Reduced BP** or associated symptoms of end-organ dysfunction (e.g. hypotonia [collapse], syncope, incontinence)
2. **Two or more of the following that occur rapidly after exposure to a** ***likely*** **allergen for that patient** (minutes to several hours): a. **Involvement of the skin-mucosal tissue** (e.g., generalized hives, itch-flush, swollen lips-tongue-uvula) b. **Respiratory compromise** (e.g., dyspnea, wheeze, bronchospasm, stridor, reduced PEF, hypoxemia) c. **Reduced BP** or associated symptoms (e.g., hypotonia [collapse], syncope, incontinence) d. **Persistent GI symptoms** (e.g., painful abdominal cramps, vomiting)
3. **Reduced BP after exposure to a** ***known*** **allergen for that patient** (minutes to several hours): a. **Infants and children:** low systolic BP (age specific) or > 30% decrease in systolic BP^a^ b. **Adults:** systolic BP < 90 mmHg or > 30% decrease from that person’s baseline

*PEF* peak expiratory flow, *BP* blood pressure, *GI* gastrointestinal

^a^Low systolic blood pressure for children is age specific and defined as: < 70 mmHg for age 1 month to 1 year; < 70 mmHg + [2 × age] for age 1–10 years; < 90 mmHg for age 11–17 years

## Diagnosis

The diagnosis of food allergy requires a detailed history and physical examination, as well as diagnostic tests such as skin prick tests (SPT) and/or food-specific serum IgE assessment. In some cases, an oral food challenge (OFC) may also be required [6, 8]. Referral to an allergist is important to confirm the diagnosis of a suspected food allergy. Patients should avoid the food in question until assessment, and an epinephrine auto-injector (EAI) should be prescribed, even if the diagnosis is uncertain [5].

### History

It is important to inquire about all suspect foods and to discuss the manner of food preparation (e.g., cooked, raw, added spices or other ingredients). Time of onset of symptoms in relation to food exposure, symptom duration and severity, as well as reproducibility of symptoms in the case of recurrent exposure should be determined. It is also important to ask about factors that can potentiate an allergic reaction, such as exercise, non-steroidal anti-inflammatory drugs, or alcohol [6, 8]. Some patients will only experience a reaction when the allergen is eaten simultaneously with one of these co-factors [24].

### Diagnostic tests

In general, diagnostic tests for food allergy (e.g., SPT, food-specific serum IgE, and OFCs) should be performed by an allergist. The SPT is a rapid, safe and sensitive method for diagnosing suspected IgE-mediated food allergy. A positive SPT appears as a wheal and flare reaction when the responsible food is applied to the skin and pricked. A positive SPT has a sensitivity of approximately 90%; however, its specificity is only around 50%. Therefore, a positive SPT alone is not sufficient for diagnosing food allergy; the patient must also have a supportive history. To minimize false positive results, over-testing with SPT should be avoided. SPT should only be done for those foods that are implicated by the patient’s history. The negative predictive value of SPT is greater than 95%. Therefore, a negative SPT generally confirms the absence of IgE-mediated reactions [6, 20]. Although less sensitive and more costly than SPTs, food-specific IgE can also be measured in serum for diagnosing food allergy, particularly if SPTs cannot be performed or are not available [6], as well as to help determine when an allergy is outgrown.

Component resolved diagnostic testing (CRD) is a relatively new method (blood test) to determine the risk or severity of allergic reactions to specific foods (e.g., peanut, hazelnuts, CM, egg, etc.). CRD can also identify cross-reactive specific components to other similar allergens from different pollen species or foods. For peanut, Ara h 8 is positive in those experiencing an oral allergy syndrome, whereas Ara h 2 is the most consistent marker for predicting peanut allergy. However, the diagnostic accuracy of a specific level of serum IgE to Ara h 2 varies between studies [25].

If the diagnosis is uncertain based on SPT and/or food allergen-specific IgE results, but there is still clinical suspicion of food allergy, an OFC may be appropriate. OFC involves gradual feeding of the suspected food with medically supervised assessment for any symptoms. In the event of symptoms, feeding is discontinued and the patient is treated where appropriate.

OFCs should only be conducted by a healthcare provider, usually an allergist, who is experienced with food allergy and anaphylaxis management, and who has established procedures for conducting these challenges [26]. In addition, OFCs must be conducted in a proper office- or hospital-based setting with resuscitation equipment. Documentation of informed consent prior to the challenge should detail that the risks and benefits of the procedure were explained to the patient or caregiver, and that these risks were understood. Healthcare providers conducting OFCs should also have an established plan for advising the patient based on the outcome of the challenge.

Other strategies that can help assist in the diagnosis of food allergy are elimination diets and food/symptom diaries. The elimination diet can be used for both the diagnosis and treatment of food allergy and requires complete avoidance of suspected foods or groups of foods for a given period of time (usually 1–2 weeks) while monitoring for an associated decrease in symptoms. It is limited by potential patient and physician bias as well as variable patient adherence to the diet. Therefore, an elimination diet should only be undertaken under the supervision of an experienced medical professional. Food/symptom diaries require the patient to keep a chronological record of all foods eaten and any associated adverse symptoms. These records may be helpful for identifying the food implicated in an adverse reaction; however, they are not usually diagnostic, particularly when symptoms are delayed or infrequent [6, 8].

## Treatment

**Fig. 2:**
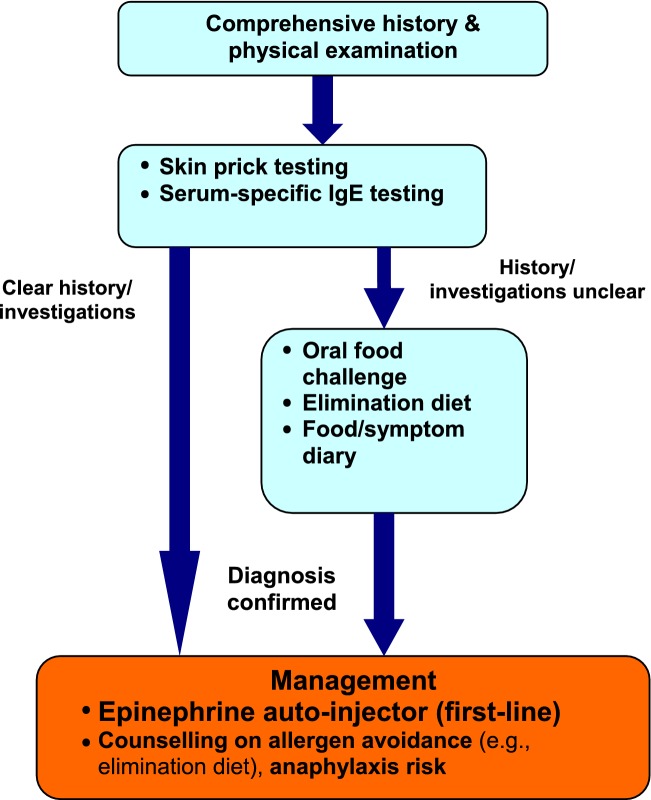
**Simplified algorithm for the diagnosis and management of food allergy**. *IgE* immunoglobulin E

A simplified algorithm for the diagnosis and management of food allergy is provided in Fig. 2.

### Food avoidance

Once a food allergy is diagnosed, strict elimination of the offending food allergen from the diet is necessary. A properly managed, well-balanced elimination diet will keep an individual free of symptoms while maintaining nutritional status. When the elimination diet is used as treatment, the relevant food should only be reintroduced once evidence exists that the food allergy has resolved [6, 8].

### Pharmacotherapy

In case of accidental exposure, the treatment of choice is epinephrine administered by intramuscular injection into the lateral thigh [6, 8]. There is currently one epinephrine auto-injector (EAI) in Canada, EpiPen^®^, which is available in two dosages (0.15 and 0.30 mg) and is prescribed according to weight. The 0.30-mg dosage should be used for those weighing 30 kg or more, and the 0.15-mg dosage for children weighing between 15 and 30 kg [27]. The American Academy of Pediatrics (AAP) recommends switching most children from the 0.15-mg dose to the 0.3-mg dose when they reach a body weight of > 25 kg [28].

These devices should be stored properly (avoiding temperature extremes) and replaced before the expiration date. More information on EAIs is available at http://www.epipen.ca. All individuals receiving emergency epinephrine must be transported to hospital immediately (ideally by ambulance) for evaluation and observation [29].

A concise, written action plan for the treatment of allergic reactions resulting from accidental exposure to the food should be developed, and copies made available to the appropriate persons (e.g., caregivers, daycare providers, teachers, employers). Examples of action plans can be downloaded at Food Allergy Canada (http://foodallergycanada.ca/resources/national-guidelines/).

Patients and their caregivers must be educated on food avoidance, the recognition and treatment of allergic and anaphylactic reactions, the appropriate use of EAIs, and how to obtain immediate medical assistance. Individuals should also be instructed to read food labels carefully, watching for hidden ingredients such as “natural flavour” or “spices” that may contain allergens, as well as “may contain” warnings. Consultation with a registered dietitian may be beneficial in this regard and may help prevent further reactions [30]. All food-allergic patients should wear medical identification, such as a MedicAlert^®^ bracelet/necklace, indicating their food allergy [5].

Food allergy is associated with a significant psychosocial burden for patients and families, which can lead to social limitations, hypervigilance and anxiety. Patients or parents with extreme anxiety or symptoms of post-traumatic stress disorder following life-threatening reactions should be referred for professional support [31].

### Food desensitization

At present, there is no treatment for food allergy beyond avoidance of the culprit food and carriage of epinephrine, however, current research is focused on food desensitization. In desensitization, patients do not react to the food allergen but are continuing to receive treatment with the food on a regular basis. With tolerance (also known as sustained unresponsiveness), patients have stopped treatment and continue not to react to the food allergen.

Oral, epicutaneous and sublingual routes of food desensitization administration have been investigated. Reported rates of patient desensitization vary from 35% to 100% (intention to treat), with much lower rates noted for sustained unresponsiveness [32]. Although commercial products are expected to become available in the near future, there are currently no approved products in Canada. Hence, these treatments are primarily available through research protocols.

#### Oral immunotherapy

In oral immunotherapy (OIT), the food is slowly introduced under medical supervision, and the dose of food increased every 2 weeks until a predefined maintenance dose is reached. With the exception of the biweekly dose escalations, daily dosing is done at home. A maintenance dose is then eaten every day to maintain desensitization. Efficacy is determined by an OFC to the food in question. While multiple randomized control trials have confirmed that OIT is often effective for inducing desensitization to various food allergens, this treatment is also associated with a risk of reactions to the food doses [33].

Most patients experience mild adverse events (e.g., oropharyngeal pruritus, GI symptoms) that resolve without treatment or with oral antihistamines. However, adverse reactions requiring epinephrine may occur during OIT. Therefore, all patients must be equipped with an EAI and an emergency action plan. Approximately 2.7% of those treated with OIT in clinical trials develop EoE [34].

Although desensitization is not a cure, studies indicate improved quality of life and less anxiety for those who have completed this process. Some allergy societies have included OIT as part of their food allergy practice guidelines, but recommend that, at present, it be restricted to experienced professionals in specialized centres [35, 36].

Recently, investigators in Australia have reported on the long-term outcomes of combined probiotic and peanut oral immunotherapy (PPOIT). These investigators had previously reported on the findings of an 18-month randomized controlled trial which found that, compared to placebo, PPOIT was effective in inducing desensitization and 2-week sustained unresponsiveness in children with peanut allergy [37]. A follow-up study, which included 48 patients from this original trial (24 from the PPOIT group and 24 from the placebo group), was designed to assess whether the previously reported benefits of PPOIT were maintained 4 years after treatment cessation [38]. The follow-up study found that 67% of subject from the PPOIT group (16 of 24) were still eating peanut 4 years after stopping study treatment, compared to 4% of subjects from the placebo group (1 of 24). A substudy of 27 participants (12 from the PPOIT group and 15 from placebo group) that agreed to undergo food challenges to assess sustained unresponsiveness, showed that 58% of participants from the PPOIT group (7 of 12) attained 8-week sustained unresponsiveness, compared with 7% of participants from the placebo group (1 of 15). These data are consistent with other clinical trials of peanut OIT since, in the 4 years of follow up, the majority of active treatment subjects continued to eat peanut, thereby maintaining their desensitization. Furthermore, the efficacy of probiotics in PPOIT cannot be determined since the original randomized trial did not include a group that received peanut OIT *without* probiotics nor a group that received probiotics alone.

#### Epicutaneous immunotherapy

In epicutaneous immunotherapy (EPIT), the food is contained in a patch which is applied to the skin. A study of peanut allergic subjects aged 4–25 years found that treatment with 250 μg peanut patches was safe and associated with a modest response after 52 weeks, with the highest responses noted in younger children [39]. An extension study that included 18 children treated with 250 μg peanut patches for 3 years revealed a trend toward better treatment responses with long-term therapy, with no decrease in adherence or increase in adverse events [40]. A 95% overall adherence rate was observed throughout the study, and no serious adverse events or epinephrine use due to therapy was reported over the 3-year study period. Most adverse events were related to the application site; skin changes were mild to moderate and decreased in both severity and frequency over time.

#### Sublingual immunotherapy

Desensitization by sublingual immunotherapy (SLIT) utilizes dissolvable tablets or liquid allergen extracts that are placed daily under the tongue and held in place for several minutes before spitting out or swallowing. Although associated with less adverse events than OIT, SLIT is generally not as efficacious [32].

#### Baked egg and cow’s milk (CM)

Studies have shown that 69–83% of CM-allergic children can tolerate baked CM, and 63–83% of egg-allergic children can tolerate baked egg [41]. The introduction of baked egg or CM has also been shown to significantly increase rates of oral tolerance to the raw forms of these foods [42, 43].

## Prevention of food allergy

### Early introduction of food

According to current guidelines, an infant with at least one first-degree relative (parent or sibling) with a history of allergic disease such as allergic rhinitis, asthma, eczema, or food allergy is at greater risk of developing food allergy [44]. Observational studies suggest that the early introduction of peanut, egg, or CM may prevent the development of allergy to these foods [45–47] (for a more detailed discussion of this topic, please see article entitled *Early Introduction of Foods to Prevent Food Allergy * in this supplement). The Learning Early About Peanut (LEAP) trial showed that the early consumption of peanut in high-risk infants (defined as those with severe eczema and/or egg allergy) reduced the development of peanut allergy by 86% by 5 years of age [48]. The Persistence of Oral Tolerance to Peanut (LEAP-On) follow-up study investigated whether participants who had consumed peanut in the primary trial would remain protected from peanut allergy after cessation of peanut consumption for 12 months [49]. The LEAP-On investigators found that the benefits of early peanut introduction persisted after 12 months of cessation of peanut consumption. Based on the LEAP findings, updated AAP-endorsed guidelines outline a new approach to reducing the risk of peanut allergy (see Table 5) [50].

**Table 5:** Summary of Addendum Guidelines 1, 2, and 3 [50]

Addendum guideline	Infant criteria	Recommendations	Earliest age of peanut introduction
1	Severe eczema, egg allergy, or both	Strongly consider evaluation by sIgE measurement and/or SPT and, if necessary, an OFC. Based on test results, introduce peanut-containing foods	4–6 months
2	Mild-to-moderate eczema	Introduce peanut-containing foods	Around 6 months
3	No eczema or any food allergy	Introduce peanut-containing foods	Age appropriate and in accordance with family preferences and cultural practices

*sIgE* serum immunoglobulin E, *SPT* skin prick test, *OFC* oral food challenge

The Enquiring about Tolerance (EAT) trial hypothesized that the early introduction of six allergenic foods (peanut, cooked egg, CM, sesame, whitefish, and wheat) in exclusively breastfed infants who were 3 months of age (early introduction group) would reduce the prevalence of food allergy by age 3 years compared to infants who were exclusively breastfed for 6 months (standard introduction group) [51]. The intention-to-treat analysis revealed a 20% reduction in the prevalence of food allergy in the early introduction group that was not statistically significant, likely because of the high rate of non-adherence to the dietary protocol.

### Use of probiotics/prebiotics

The World Allergy Organization/McMaster University Guidelines for Allergic Disease Prevention (GLAD-P) provide graded recommendations on the use of probiotics and prebiotics for allergy prevention based on current available evidence [52, 53]. GLAD-P recommends the use of probiotics in pregnant and breastfeeding women whose children and infants are at high risk for developing allergy (conditional recommendation, very low quality evidence) [52]. Ultimately, the use of probiotics should be individualized, and further studies are needed to evaluate their efficacy in preventing other types of allergy, as well as the differences among the strains of the same species of probiotic bacteria. The guidelines do not to provide a recommendation regarding prebiotic supplementation in pregnancy or during breastfeeding due to the lack of experimental and observational studies [53].

## Prognosis

The prognosis of food allergy is complex and dependent on the particular food. Although most infants and young children outgrow allergies to CM, egg, soy and wheat, there is evidence that an increasing number of children may not outgrow allergies to CM and egg until their teenage years [12, 13]. Children should be re-evaluated by their allergist at regular intervals to determine whether clinical tolerance has developed. In most cases, allergy to peanut, tree nuts, fish, and shellfish is lifelong.

## Conclusions

IgE-mediated food allergy is an important clinical problem of increasing prevalence. Assessment by an allergist is essential for appropriate diagnosis and treatment. Diagnosis is based on a careful history and diagnostic tests, such as SPT, food-specific serum IgE testing (where appropriate) and, if indicated, OFCs. The mainstay of treatment is avoidance of the responsible food(s), and timely administration of epinephrine for allergic reactions. Current research on treatment is focused on food desensitization. Further insights into the pathophysiology of food allergy and anaphylaxis will lead to the development of improved methods for prevention, diagnosis, and management.

## Key take-home messages

- Food allergy is defined as an adverse immunologic response to a food protein.
- Referral to an allergist is important for appropriate diagnosis and treatment.
- Diagnosis of a food allergy requires a detailed history and diagnostic tests, such as SPT and/or food-specific serum IgE measurement; in some cases, OFCs may also be required.
- Management of food allergy involves avoidance of the responsible food(s) and injectable epinephrine.
- For patients with systemic symptoms, the treatment of choice is epinephrine administered by intramuscular injection into the lateral thigh.
- Data suggests that it may take longer to “outgrow” allergies to CM and egg than previously reported. Allergy to peanut, tree nuts, fish, and shellfish is usually life-long.

## Abbreviations

IgE: immunoglobulin E
SPT: skin prick tests
OFC: oral food challenge
CM: cow’s milk
AD: atopic dermatitis
EoE: eosinophilic esophagitis
GI: gastrointestinal
EAI: epinephrine auto-injector
OIT: oral immunotherapy
EPIT: epicutaneous immunotherapy
SLIT: sublingual immunotherapy
LEAP: Learning Early About Peanut study
LEAP-On: Persistence of Oral Tolerance to Peanut follow-up study
EAT: Enquiring About Tolerance study
GLAD-P: World Allergy Organization-McMaster University Guidelines for Allergic Disease Prevention
CRD: component resolved diagnostic testing
AAP: American Academy of Pediatrics
PPOIT: combined probiotic and peanut oral immunotherapy
